# Photocurable Carbon Nanotube/Polymer Nanocomposite for the 3D Printing of Flexible Capacitive Pressure Sensors

**DOI:** 10.3390/polym15244706

**Published:** 2023-12-14

**Authors:** Jia-Wun Li, Ho-Fu Chen, Peng-Han Huang, Chung-Feng Jeffrey Kuo, Chih-Chia Cheng, Chih-Wei Chiu

**Affiliations:** 1 Department of Materials Science and Engineering, National Taiwan University of Science and Technology, Taipei 10607, Taiwan; 2 Graduate Institute of Applied Science and Technology, National Taiwan University of Science and Technology, Taipei 10607, Taiwan

**Keywords:** 3D printing, photocuring, carbon nanotubes, sensing element, capacitive pressure sensor

## Abstract

A photocurable resin/carbon nanotube (CNT) nanocomposite was fabricated from aligned CNTs in an acrylic matrix. The conductivity of the nanocomposite increased rapidly and then stabilized when the CNT content was increased up to and beyond the percolation threshold. Various structures were created using a digital light processing (DLP) 3D printer. Various polymeric dispersants (SMA-amide) were designed and synthesized to improve the CNT dispersion and prevent aggregation. The benzene rings and lone electron pairs on the dispersant interacted with aromatic groups on the CNTs, causing the former to wrap around the latter. This created steric hindrance, thereby stabilizing and dispersing the CNTs in the solvent. CNT/polymer nanocomposites were created by combining the dispersant, CNTs, and a photocurable resin. The CNT content of the nanocomposite and the 3D printing parameters were tuned to optimize the conductivity and printing quality. A touch-based human interface device (HID) that utilizes the intrinsic conductivity of the nanocomposite and reliably detects touch signals was fabricated, enabling the free design of sensors of various styles and shapes using a low-cost 3D printer. The production of sensors without complex circuitry was achieved, enabling novel innovations.

## 1. Introduction

Due to rapid technological advancement [1], many products become obsolete soon after they are released. Therefore, short product development cycles, low costs, high quality, customizability, and low inventory levels have become necessary for commercial success [2]. Additive manufacturing (AM) is a layer manufacturing technique that has emerged and developed in this context [3,4]. From 1990 to 2010, AM was widely used for rapid prototyping (RP) [5,6]. Unlike conventional subtractive manufacturing, AM creates 3D models by additively stacking layers [7,8], which facilitates the creation of customized, complex, and detailed structures [9]. AM is especially competitive in the medical [10], transportation [11], and aerospace industries [12], as it is well suited for the manufacturing of customized and light-weight microcellular structures [13]. The emergence of the maker movement in 2010 led to the popularization of a new low-cost, desktop-based AM technique called 3D printing (or personal prototyping) [14], which lowered the barrier to entry for AM [15]. In 2009, ASTM International formally divided 3D printing into seven major categories, which include direct ink writing (DIW) [16], fused deposition modeling (FDM) [17], and vat photopolymerization (VP) [18]. VP can be further subdivided into stereolithography (SLA) [19], digital light processing (DLP) [20], and liquid crystal display (LCD) [21] 3D printing. DLP 3D printing is popular due to its speed, high precision, and low resin usage. This type of printing is based on photopolymerization, where a liquid resin is irradiated with UV light of a specific wavelength, causing it to solidify rapidly into a shape. Because this solidification occurs in an aqueous environment, unlike other 3D printing techniques, DLP can be used to create hollow or porous structures without using supports [22,23,24]. The advantage of 3D printing is its flexibility. In addition to creating products of various shapes, DLP can also be used to construct complex 3D structures. This flexibility makes it highly competitive for medical and biotechnological applications [25]. For instance, 3D printing can be used to create customized medical devices or human tissue scaffolds [26] for medical purposes or to create cell culture devices for biotechnological applications [27]. In addition, 3D printing can be used to create custom sensors for a wide range of applications [28], thus creating new opportunities in many areas.

In summary, DLP 3D printing is a highly efficient, precise, and flexible technique that has broadened the horizon of possibilities in many areas [29,30,31]. However, the 3D printing of a capacitive sensor generally requires the use of a photocurable resin with some degree of conductivity [32,33,34]. Wang et al. [35] uniformly dispersed carbon black nanoparticles and NaCl in a TPU elastomeric matrix, producing a printable ink. They successfully utilized 3D printing with FDM technology to fabricate a soft game controller. However, the intense black color of carbon black may be disadvantageous in DLP printing. Carbon nanotubes (CNTs) are helical microtubules of graphitic carbon that form 1D structures with diameters in the nanometer range. At the microscopic level, CNTs may be viewed as seamless cylinders (with single or multiple concentric shells) formed by rolling up a honeycomb lattice of carbon hexagons [36]. Due to their outstanding electrical, thermal, and mechanical properties, various multifunctional polymer composites have been developed using CNTs [37]. Conductive polymer nanocomposites are already being used in electromagnetic interference shielding [38], anti-static coatings [39], flexible electronics [40], and strain sensors [41]. Therefore, CNTs are a judicious choice as an additive for conductive photocurable resins [42]. The mechanisms underlying the electrical conductivity in conductive nanocomposites include inter-aggregate conduction and field-emission and electron tunneling. These mechanisms can either be Ohmic (occurring through direct contact) or based on charge separation [43], depending on the distribution of the filler. In a polymer matrix, CNTs will create a so-called “percolation network” when their concentration exceeds the percolation threshold [44]. Upon the application of an electric field, electrons will move through this percolation network, which causes the conductivity to increase by multiple orders of magnitude. Because the formation of the percolation network depends on the dispersion of the CNTs, aggregation will make it difficult to form such a network [45]. However, it is possible to achieve high conductivities at lower loadings by obtaining a heterogeneous distribution of CNTs through the formation of a segregated structure. In these structures, certain parts of the polymer matrix will have high concentrations of CNTs, which results in the formation of conductive nanocomposites with low CNT loadings [46]. Therefore, the dispersion properties of the CNTs are an important factor for conductive nanocomposites. The CNTs can achieve conductivity with lower loading quantities compared to carbon black due to the utilization of ultraviolet light for resin curing in 3D printing DLP technology. Simultaneously, this 3D printing process ensures the complete curing of CNTs/resin.

In this study, to expand the application range of 3D printing and add value to 3D printed products, a 3D-printed nanocomposite was created by combining an acrylic polymer matrix with a CNT nanofiller. By exploiting the characteristics of this nanocomposite, the physical and mechanical properties of 3D-printed objects were improved, and a conductive polymer nanocomposite was ultimately successfully created. Furthermore, a complex 3D structure was printed by fully utilizing the advantages of 3D printing. To improve the CNT dispersion in the resin, polymeric dispersants designed by our group were utilized, and the CNT dispersion was maximized by selecting the optimal type of dispersant and the optimal dispersant ratio. The dispersants, CNTs, and photocurable resin were combined to create a photocurable CNT/polymer nanocomposite. Finally, the amount of CNTs added and the printing parameters were tuned to optimize the conductivity and product quality.

## 2. Materials and Methods

### 2.1. Materials

The photocurable urethane acrylate oligomer (trifunctional aliphatic urethane acrylate, Photomer 6184) and photocurable aliphatic trifunctional acrylate (trimethylolpropane propoxylate triacrylate, Photomer 4072) were purchased from Henkel, Düsseldorf, Germany). The photoinitiator (Irgacure 784) was purchased from Ciba, Basel, Switzerland. The CNTs (A-MWCNT08, diameter: 8 nm, length: 100–200 nm, specific surface area: 400–700 m^2^/g) were purchased from Conjutek Co., New Taipei City, Taiwan. Styrene maleic anhydride (SMA) copolymer (SMA^®^ EF80) was purchased from Yuang Hong Corporation, Taipei, Taiwan. Jeffamine-M1000 and Jeffamine-D2000 were purchased from Huntsman Chemical Co., Los Angeles, CA, USA.

### 2.2. Synthesis of Polymeric Dispersants (SMA-Grafted Polyetheramine SMA-M1000 and SMA-D2000)

The SMA-M1000 and SMA-D2000 polymeric dispersants were prepared according to the procedures outlined in Ref. [47]. The relevant syntheses are shown in Figures S1 and S2 in the Supplementary Materials. First, SMA and M1000 (or D2000) were vacuum-dried for 3 h at 100 °C. Thereafter, 0.01 mol of SMA was dissolved in 10 mL of tetrahydrofuran (THF) in a 1-neck flask, and 0.015 mol of M1000 (or D2000) was slowly added over 3 h with stirring at 25 °C. To ensure that the reaction was completed, excess M1000 (or D2000) was added. During this process, the amine reacted with SMA to form amic acid groups. Upon the completion of the reaction, unreacted M1000 (or D2000) was removed from the mixture, and the reaction system was heated at 60 °C for 12 h to obtain the SMA-M1000 (or SMA-D2000) copolymer.

### 2.3. Preparation of CNT Dispersions

Two dispersants, SMA EF80-M1000 and SMA EF80-D2000, were used to prepare the CNT dispersions. The CNTs and dispersants were added to the THF solvent in the required ratio to create a 0.05 wt% solution. First, a pre-dispersion step was performed by ultrasonicating the solution for 15 min. The CNTs were dispersed via ultrasonication using the following parameters: amplitude: 10, pulse-on time: 10 s, pulse-off time: 3 s, and process time: 30 min. Different CNT/dispersant ratios were also evaluated (1:0, 1:0.5, 1:1, 1:1.5, and 1:2) to examine how the CNT/dispersant ratio affects the particle size and aggregation in the solution with the help of transmission electron microscopy (TEM), dynamic light scattering (DLS) spectroscopy, and UV-vis transmittance spectroscopy. The optimal CNT/dispersant ratio was determined using the experimental results.

### 2.4. Preparation of Photocurable Resins Containing Dispersed CNTs

Mechanical stirring was used to prepare the photocurable resin. First, the CNT dispersion and Photomer 4072 were mixed in a high-shear homogenizer at 6000 rpm to prepare uniformly dispersed CNTs. The THF was removed using a vacuum concentrator. Finally, Photomer 6184 and the photoinitiator were added to the solution and stirred at 6000 rpm for 1 h to prepare the photocurable resin/CNT nanocomposite. Because the mechanical stirring created small bubbles in the resin that would have severely deteriorated the printing quality, the bubbles were removed via ultrasonication and vacuum degassing prior to the printing process.

### 2.5. Characterization and Instruments

Fourier-transform infrared (FT-IR) spectroscopy (with a FTS-1000, Hopkinton, MA, USA) was used to monitor the SMA-amide reaction. Scans were performed in the 500–4000 cm^−1^ range, with each FT-IR spectrum being the average of 32 scans. A Zetasizer Nano-ZS90 instrument (Malvern Panalytical, Malvern, UK) was used to monitor the particle size by scanning in the 1–10,000 nm range, with each particle size spectrum being the average of 10 scans. A Shimadzu UV-2450 (Kyoto, Japan) UV-vis spectrometer was used to measure the changes in the transmittance of the CNT dispersion at 550 nm, and each transmittance result is an average of five samples. TEM (Zeiss EM-902A; Oberkochen, Germany) was used to characterize the dispersion of the CNTs. First, a 1 wt% sample was prepared, a fixed volume of which was then deposited onto a carbon-coated copper grid for TEM examination. Differential scanning calorimetry (DSC) was performed using a Perkin Elmer (Waltham, MA, USA) DSC-6000 instrument. The sample was heated to 200 °C at a rate of 10 °C/min while scanning to monitor the solidification of the photocurable resin. An Anton Paar rotational rheometer was used to analyze the changes in the viscosity of the resin during solidification with a shear rate of 100/s and a temperature of 25 °C. Each viscosity result is an average of three samples. A World Meterology MTS-370 universal testing machine (MTS Systems Corp., Eden Prairie, MN, USA) was used to assess the mechanical properties of dumbbell-shaped specimens of the photocurable resin according to the ASTM D638 method [48]. The electrical resistivity of the photocurable resin was measured using a four-point probe (Mitsubishi MCP-T600 low resistivity meter; Mitsubishi, Tokyo, Japan), and each resistivity result is an average of five samples. The surface morphology and profile of the photocurable resin samples were examined using a JSM-6500F field-emission scanning electron microscope (FE-SEM) (JEOL Ltd., Tokyo, Japan), where the samples were coated with a layer of platinum prior to the FE-SEM examination.

## 3. Results and Discussion

### 3.1. Synthesis of SMA-Amide Polymers and Their Efficacy as CNT Dispersants

**Figure 1:**
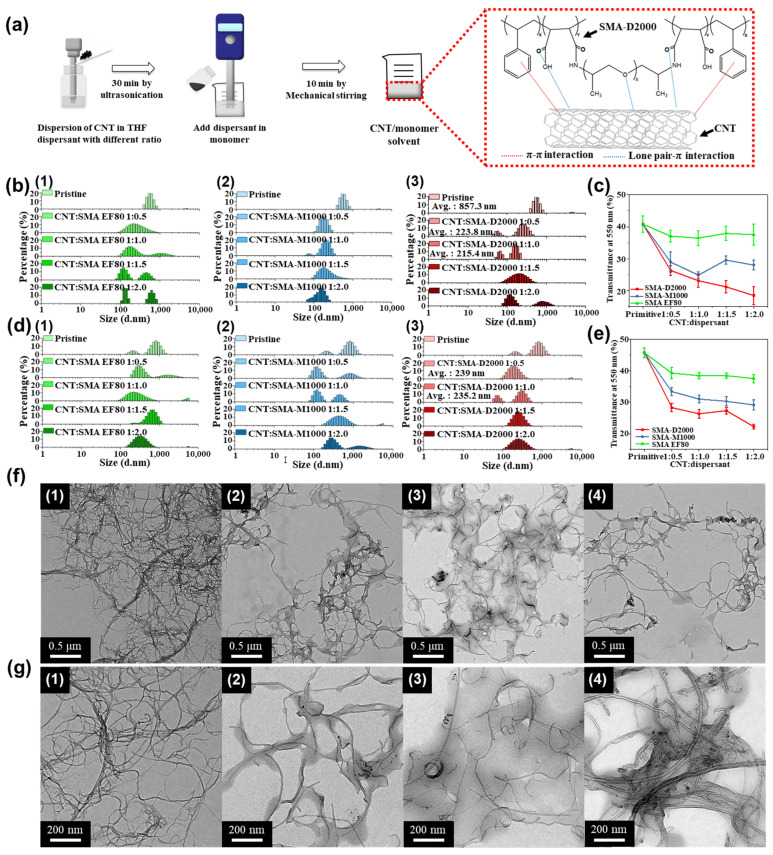
(**a**) Mechanism by which SMA-amide disperses CNTs. (**b**) Particle size analysis of the CNT/polymeric dispersant solutions on the 0th day: (1) SMA EF80, (2) SMA-M1000, (3) SMA-D2000. (**c**) Transmittance of the CNT/polymeric dispersant solutions at 550 nm on the 0th day. (**d**) Particle size analysis of the CNT/polymeric dispersant solutions on the 5th day: (1) SMA EF80, (2) SMA-M1000, (3) SMA-D2000. (**e**) Transmittance of the CNT/polymeric dispersant solutions at 550 nm on the 5th day. (**f**,**g**) TEM micrographs of CNT/polymeric dispersant solutions at different magnifications with (1) no dispersant, (2) SMA EF80, (3) SMA-M1000, and (4) SMA-D2000.

FT-IR was used to confirm the formation of the polymeric dispersants (Figures S3 and S4 in the Supplementary Materials). The characteristic absorptions of maleic anhydride occur in the 1750–1870 cm^−1^ range, and the absorptions corresponding to N-H substitution in the SMA-amide polymers are located in the amide I (1670–1870 cm^−1^) and amide II (1510–1550 cm^−1^) bands. The disappearance of the absorptions corresponding to maleic anhydride (1854–1860 cm^−1^ and 1772–1779 cm^−1^) and the appearance of the amide I (1679 cm^−1^ and 1726 cm^−1^) and amide II (1492 cm^−1^) absorptions confirm that SMA was successfully grafted onto polyetheramine (PEA) to form the SMA-amide polymers. Because the SMA copolymer is lipophilic, it can only dissolve in non-polar, organic solvents. Therefore, when SMA is mixed with polar solvents like ethanol, emulsification will occur. PEA, on the other hand, has hydrophilic ends on its molecular chains. These chains allow PEA to dissolve in both polar and non-polar solvents. Due to the hydrophilic and lipophilic segments on its molecular chains, PEA is easily dissolved and processed, which makes it suitable for most dispersants, as shown in Table S1 in the Supplementary Materials. As shown in Figure 1a, the benzene groups and lone pairs on the SMA-amide polymeric dispersant can interact with the aromatic groups on the CNTs via π–π bonds and lone pair–π stacking interactions, respectively. This causes the PEA chains to wrap around the CNTs, creating steric hindrance that stabilizes and disperses the CNTs in the solution. Therefore, the mechanism by which polymeric dispersants improve the dispersion and stability of the CNTs involves the formation of covalent bonds between the aromatic groups on the CNTs and benzene rings on the lipophilic segments of the SMA-amide polymer, which creates steric hindrance that stabilizes and disperses the CNTs. CNT dispersions were prepared by adding the CNTs and polymeric dispersants to THF in various ratios (all with a total concentration of 0.05 wt%), followed by ultrasonication. Photographs of the resulting solutions are shown in Figure S5 in the Supplementary Materials. The addition of SMA-D2000 improved the dispersion of the CNTs, as the CNT residue on the bottle decreased and became finer. Different polymeric dispersants were added to the CNT suspension and were observed on the 0th and 5th days after ultrasonic dispersion (Figures S6 and S7 in the Supplementary Materials). The bottle without dispersant had many particles on its walls and CNT aggregates that settled at the bottom. The SMA-amide polymeric dispersants were more effective than SMA (EF80). As CNTs tend to aggregate without dispersants, various dispersed CNT solutions were prepared by mixing the CNTs with a polymeric dispersant in THF in varying mass ratios (1:0, 1:0.5, 1:1, 1:1.5, and 1:2). A particle size analyzer was used to characterize each solution; the particle size distributions are shown in Figure 1b. The “pristine CNT” solution (1:0 solution) had an average particle size of 857 nm, indicative of aggregation. The addition of all dispersants caused a leftward shift of the average particle size from 857 nm to <300 nm; when the CNT/dispersant mass ratio reached 1:1, the average particle size (across all dispersants) was only 215.4 nm. Hence, the CNT aggregates were already partially dispersed at this ratio. The UV-vis data acquired at 550 nm are shown in Figure 1c. Each transmittance result is an average of five samples. In situations characterized by good dispersibility, nanotubes exhibit effective dispersion within the solution. The uniformly dispersed nanotubes act as a barrier, blocking light and consequently leading to a reduction in transmittance. Conversely, when nanotubes undergo aggregation or precipitation, the fewer carbon nanotubes in the suspension allow light to penetrate the solution more easily [28]. The solution without dispersants had a transmittance of 40.8, and the transmittance of the solutions containing SMA EF80 did not decrease significantly, irrespective of the mass ratio. The lowest transmittance was observed for the solutions containing SMA EF80-D2000 with a CNT/dispersant mass ratio of 1:1. Further increases in the dispersant content led to significant decreases in the transmittance. The particle sizes of the solutions were analyzed after leaving the solutions undisturbed for five days (Figure 1d). The particle size distributions of some of the solutions showed shifts to higher particle sizes, as the dispersant failed to provide enough steric hindrance to prevent aggregation and settling. However, the particle size distributions of the solutions containing SMA-D2000 showed virtually no change. Following calculations, it was determined that the CNT/SMA-D2000 ratio was maintained at 1:1. After allowing the solution to stand undisturbed for five days, the average particle size showed a shift from the initial value of 215.4 nm to 235.2 nm, suggesting that there was no significant alteration in the average particle size after the five-day period of standing undisturbed, which indicates that this dispersant was able to maintain and stabilize the CNT dispersion. The transmittance (at 550 nm) of the suspensions that were left to sit for five days was also analyzed (Figure 1e). The transmittance of the suspensions without any dispersant or SMA EF80 (SMA without PEA grafting) increased significantly due to settling and aggregation. TEM was used to examine the dispersion of the CNTs in each solution (Figure 1f,g). The CNTs formed knot-like aggregates in the suspension without dispersant. Dispersant addition significantly reduced this form of aggregation. Furthermore, the steric hindrance caused by the dispersants created distance between the CNTs, which prevented aggregation and stabilized the CNT dispersion.

### 3.2. Preparation of Photocurable Resin

**Figure 2:**
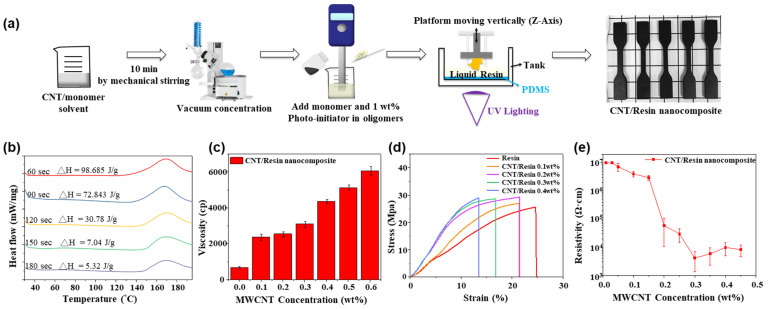
(**a**) Preparation of CNT/resin nanocomposites. (**b**) DSC analysis of CNT/resin nanocomposites with different photocuring times. (**c**) Resin viscosity versus CNT content. (**d**) Stress-strain curves of CNT/resin nanocomposites with different CNT contents. (**e**) Nanocomposite resistivity versus CNT content.

First, a 1:1 CNT/SMA-D2000 solution was added to the resin before solidification in various mass ratios. After dispersing the mixture via ultrasonication, THF was removed in a vacuum concentrator. The photoinitiator was then added with stirring. Finally, the resin was photocured via 3D printing. A flow chart of this process is shown in Figure 2a. The photoinitiator is a critical component of the photocurable resin, as it determines the photocuring time, reaction time, and maximum absorption wavelength of the resin. Because each photoinitiator has a different maximum absorption wavelength, small amounts of the photoinitiator (0.1, 0.01, and 0.001 wt%) were dissolved in THF to determine the absorption range via UV-vis spectroscopy (see Figure S8 in the Supplementary Materials). The photoinitiator absorbed between 250 nm and 550 nm. Because the 3D printer used in this study had a 405 nm light source, the photoinitiator and the 3D printer were compatible. Because CNTs were added to the photocurable resin, some of the light emitted by the light source of the 3D printer was absorbed by the CNTs, which could have led to incomplete curing and thus printing failure. To ensure the consistent quality of the printed objects, prior to 3D printing, nanocomposites photocured for different times were analyzed via DSC. If the photocuring time was too short, the cross-linkages were not fully formed, which led to exothermic peaks in the DSC curve, the area of which could be calculated using a formula, as shown in Figure 2b. From these tests, the photocuring time was determined to be 240 s/layer for the first five layers and 180 s/layer for subsequent layers. Because the addition of CNTs may also reduce the precision of the layer height setting, three-layer objects were printed with three different layer height settings (30, 40, and 50 µm), and FE-SEM was used to examine the profile of each object (Figure S9 in the Supplementary Materials). The results reveal that when the thickness of the first layer was set to 40 μm or 50 μm, the initial layer thickness remained consistent as per the settings. However, during the printing of the second and third layers, samples with a thickness setting of 30 μm produced layers of equal thickness. For samples with a thickness setting of 40 μm, a slight reduction in thickness was observed, while samples with a thickness setting of 50 μm exhibited a more pronounced reduction in thickness. This variation may be attributed to machine or parameter-related errors. Nevertheless, regardless of the thickness setting, the printer was capable of producing complete and intact products. However, CNT addition may also increase the viscosity of the resin. Therefore, a rotational rheometer was used to check the effects of CNT addition on the resin viscosity, as shown in Figure 2c. Each viscosity result is an average of three samples. The resin viscosity increased with increases in the CNT content. Because it is necessary for the resin to reflow into the small gap between the printed object and the nozzle during 3D printing, an excessively high viscosity (>4500 cp) would result in bubbles inside the object, which would lead to printing failure. Therefore, the conductivity and flowability must be balanced. To this end, the CNT content was set to 0.4 wt%. A universal testing machine was used to measure the mechanical strength and elasticity of the objects printed using the CNT/resin nanocomposite. The strain–stress curves of the carbon nanotube composite material without adding dispersants are shown in Figure S10 in the Supplementary Materials. Compared with the general photocured resin, the stress did not increase significantly, but the strain decreased by about 60%. The decrease in strain is attributed to the agglomeration caused by the addition of carbon nanotubes and phase separation from the acrylic base material. The results are presented in Figure 2d after adding dispersant. The addition of the dispersants resulted in adequate CNT dispersion in the resin. Compared to ordinary photocurable resin, the ultimate stress and strain of the nanocomposite were 16% higher and 23% lower, respectively. Therefore, CNT addition gave the specimen significantly more rigidity because the randomly distributed CNTs increased the interlayer adhesion, which improved the mechanical properties of the material. SEM was used to observe the fracture surface of the nanocomposite after tensile failure, as shown in Figure S11 in the Supplementary Materials. The CNT-free resin had a relatively even fracture surface, whereas the fracture surface of the resin with 0.3 wt% CNTs had many more scratches and wrinkles. This is because the randomly distributed CNTs hindered the movement of the molecular chains in the polymer matrix, which then increased the tensile strength. Finally, the dependence of the conductivity of the photocurable resin on the CNT content was investigated using a four-point probe (Figure 2e). Each resistivity result is an average of five samples. The results reveal that when the added amounts of carbon nanotubes were 0.01 wt% and 0.03 wt%, the resistance values exceeded the detection range of the four-point probe, surpassing 1.0 × 10^7^ Ω·cm. For added amounts of carbon nanotubes at 0.05, 0.10, 0.15, 0.20, 0.25, 0.30, 0.35, 0.4, and 0.45 wt%, the resistances were 7.2 × 10^6^ ± 2.3 × 10^6^, 4.2 × 10^6^ ± 9.1 × 10^5^, 2.9 × 10^6^ ± 5.5 × 10^5^, 5.9 × 10^4^ ± 4.8 × 10^4^, 3.0 × 10^4^ ± 1.6 × 10^4^, 4.2 × 10^3^ ± 2.8 × 10^3^, 5.9 × 10^3^ ± 3.6 × 10^3^, 10 × 10^4^ ± 4.9 × 10^3^, and 8.3 × 10^3^ ± 3.6 × 10^3^ Ω·cm, respectively. The nanocomposites with CNT contents lower than 0.05 wt% were found to be insulators. The resistivity became measurable from 0.05 wt% CNT onwards, which indicates that a percolation network began to form at 0.05 wt%. At this point, the resistivity decreased rapidly with increases in the CNT content. With 0.3 wt% CNT, the resistivity declined to 4.2 × 10^3^ ± 2.8 × 10^3^ Ω·cm. Further increasing the CNT content only resulted in marginal decreases in the resistivity. Then, 3D printing was used to create conductive objects in various shapes, and a voltage was passed through these objects to illuminate an LED, as shown in Figure S12 in the Supplementary Materials.

### 3.3. Fabrication of a Capacitive Touch Keyboard Using the CNT/Resin Nanocomposite

**Figure 3:**
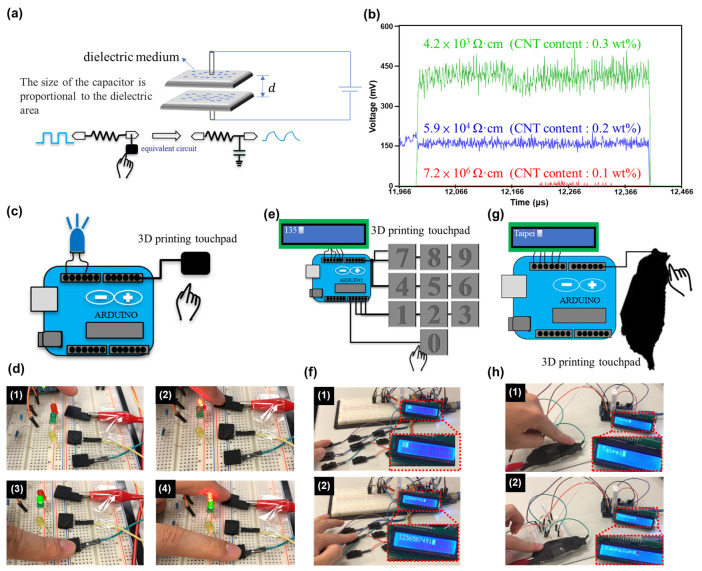
(**a**) Schematic of the capacitive sensor. (**b**) Effects of resistivity on the signal from the sensor. (**c**) Schematic of capacitive sensor connected to an LED. (**d**) Output of LED-connected capacitive sensor: (1) sensors not touched; (2) red LED touched; (3) green LED touched; (4) red and green LEDs touched at the same time. (**e**) Schematic of 3D-printed touchpad keyboard connected to LCD screen. (**f**) Output of touchpad keyboard: (1) capacitive sensor number ‘1’ touched; (2) result at the end of the test. (**g**) Schematic of 3D-printed map of Taiwan. (**h**) Output from touching the 3D-printed map: (1) result of touching the location corresponding to Taipei City; (2) result of touching the location corresponding to Kaohsiung City.

Previously, we succeeded in creating a conductive nanocomposite by adding CNTs to a photocurable resin. Here, we chose to fabricate a capacitive sensor using this nanocomposite via 3D printing. Capacitive sensing is used in a variety of sensors, including displacement, position, humidity, and acceleration sensors. Many human interface devices (HIDs) also use capacitive sensing. In the present HID device, an Arduino board was connected to the capacitive sensor on two ends, with one end outputting a pulse signal and the other end set to receive the signal. When the sensor was not touched, the pulse signal was received in its original form by the receiving end. When the sensor was touched, a capacitor formed between the finger and the conductor (which was equivalent to connecting a capacitor to the circuit), and the signal was only received after a delay. A formula was then used to compare the input and output signals to determine whether a person had touched the sensor, as shown in Figure 3a. Because the amplitude of the signal depended on the resistivity of the sensor, samples with different resistivities were prepared (4.2 × 10^3^ ± 2.8 × 10^3^, 5.9 × 10^4^ ± 4.8 × 10^4^, and 4.2 × 10^6^ ± 9.1 × 10^5^ Ω·cm), and the changes in the signal amplitude were observed (Figure 3b). The signal amplitude increased with decreasing resistivity, and the signal was virtually undetectable when the resistivity was 4.2 × 10^6^ ± 9.1 × 10^5^ Ω·cm. Hence, to facilitate signal reception, a deliberate selection of the carbon nanotube concentration of 0.3 wt% was made for subsequent applications. With the conductive properties of CNT/resin nanocomposites, combined with the flexibility of 3D printing, it is easy to create customized sensors. We wrote an Arduino program for custom sensors. (See Figure S13 in the Supplementary Materials. Capacitive sensor refers to the pulse-signal transmitting and receiving pins, while ‘Long Capacitive Sensor’ is the digitalized value of the detected signal.) By utilizing the advantages of 3D printing (and the Arduino code above), a prototype capacitive sensor was preliminarily fabricated and attached to LEDs, as shown in Figure 3c,d. Here, the program compared the output and inputs of the Arduino board and thus determined whether a finger had touched the capacitive sensor; when a finger touched the sensor, the corresponding LED would turn on. A digital keyboard was thus constructed based on 3D printing and was attached to an LCD screen (see Figure 3e,f and Video S1 in the Supplementary Materials). When a number was touched on the keyboard, it also appeared on the LCD screen. This device is immune to mechanical wear, and its appearance can be customized according to the designer’s preferences. Finally, a map of Taiwan was printed using the 3D printer, and each city was given a code, as shown in Figure 3g,h and Video S1 in the Supplementary Materials. When Taipei City was touched on the map, “Taipei” appeared on the LCD screen. Likewise, when Kaohsiung City was touched on the map, “Kaohsiung” appeared on the screen.

## 4. Conclusions

Aligned CNTs were mixed with a photocurable resin, and a polymeric dispersant (SMA-amide) designed by our group was used to disperse the CNTs to improve the physical and mechanical properties of the material. This resulted in the creation of a conductive polymer nanocomposite. The successful synthesis of the polymeric dispersants was confirmed via FT-IR analysis. The CNT dispersion was analyzed using DLS, UV-vis transmittance, and TEM to find the most suitable polymeric dispersant for the nanocomposite. Based on transmittance and particle size analyses, SMA-amide dispersants are effective in improving the dispersion of the CNTs in the THF solvent. The transmittance was lowest (optimal) when the SMA-D2000 dispersant was used in a 1:1 mass ratio with the CNTs, and the DLS analysis showed that the average particle size of the CNTs at this ratio was 215.4 nm. According to the stress-strain curves obtained via tensile testing, the nanocomposite with 0.3 wt% dispersant had the best mechanical performance among the tested specimens. Compared to an ordinary photocurable resin, the ultimate stress of this nanocomposite was 16% higher, and the strain was 23% lower. Finally, this material was used to 3D print a touch-based HID. The nanocomposite sensor fabricated in this study had a resistivity of 4.2 × 10^3^ ± 2.8 × 10^3^ Ω·cm and could reliably detect touch signals.

## Data Availability

The data presented in this study are available on request from the corresponding author.

## Supplementary Materials

The following supporting information can be downloaded at https://www.mdpi.com/article/10.3390/polym15244706/s1. Figure S1: Synthesis of SMA-M1000 polymeric dispersant; Figure S2: Synthesis of SMA-D2000 polymeric dispersant; Figure S3: FT-IR analysis of M1000, SMA EF80, and SMA-M1000; Figure S4: FT-IR spectra of D2000, SMA EF80, and SMA-D2000; Figure S5: Photographs of CNT dispersion before and after adding polymeric dispersants; Figure S6: Photographs of CNT/polymer dispersions immediately after preparation: (1) without dispersant, (2) EF80, (3) M-1000, and (4) SMA-M1000; Figure S7: Photographs of CNTs/polymer dispersions five days after preparation: (1) without dispersant, (2) EF80, (3) M-1000, and (4) SMA-M1000; Figure S8: UV-vis spectrum of the photoinitiator; Figure S9: FE-SEM observation of the cross-section of 3D-printed objects with different layer heights: (1) 30 μm, (2) 40 μm, and (3) 50 μm; Figure S10: Stress-strain curves of CNT/resin nanocomposites without dispersant; Figure S11: FE-SEM micrographs of the fracture surface of CNT/resin nanocomposites after tensile failure. (1) Pure resin (without CNT); (2) nanocomposite with 0.3 wt% CNT; (3) magnified view of an area in (2); Figure S12: Demonstration of the conductivity of an object fabricated via DLP 3D-printing with the developed photocurable resin/CNT nanocomposite; Figure S13: Arduino code for the capacitive pressure sensor; Table S1: Solubility of the polymeric dispersants in different solvents; Video S1: A video showing a demonstration of 3D-printed touchpad keyboard connected to LCD screen and 3D-printed map of Taiwan using carbon nanotube/polymer nanocomposite as capacitive pressure sensors.

## References

[1] Huang C.Y., Chiu C.W. (2021). Facile fabrication of a stretchable and flexible nanofiber carbon film-sensing electrode by electrospinning and its application in smart clothing for ECG and EMG monitoring. ACS Appl. Electron. Mater..

[2] Cui W., Yang Y., Di L., Dababneh F. (2021). Additive manufacturing-enabled supply chain: Modeling and case studies on local, integrated production-inventory-transportation structure. Addit. Manuf..

[3] Pillai S., Upadhyay A., Khayambashi P., Farooq I., Sabri H., Tarar M., Lee K.T., Harb I., Zhou S., Wang Y. (2021). Dental 3D-printing: Transferring art from the laboratories to the clinics. Polymers.

[4] Andrearczyk A., Konieczny B., Sokołowski J. (2021). Additively manufactured parts made of a polymer material used for the experimental verification of a component of a high-speed machine with an optimised geometry—Preliminary research. Polymers.

[5] Verbeeten W.M., Arnold-Bik R.J., Lorenzo-Bañuelos M. (2021). Print velocity effects on strain-rate sensitivity of acrylonitrile-butadiene-styrene using material extrusion additive manufacturing. Polymers.

[6] Gooding J.J. (2022). Developing chemical sensors that employ consumer electronics has pitfalls as well as rewards. ACS Sens..

[7] Jandyal A., Chaturvedi I., Wazir I., Raina A., Haq M.I.U. (2022). 3D printing–A review of processes, materials and applications in industry 4.0. Sustain. Oper. Comput..

[8] Jadhav A., Jadhav V.S. (2022). A review on 3D printing: An additive manufacturing technology. Mater. Today Proc..

[9] Le-Bail A., Maniglia B.C., Le-Bail P. (2020). Recent advances and future perspective in additive manufacturing of foods based on 3D printing. Curr. Opin. Food Sci..

[10] Arefin A.M., Khatri N.R., Kulkarni N., Egan P.F. (2021). Polymer 3D printing review: Materials, process, and design strategies for medical applications. Polymers.

[11] Lei D., Yang Y., Liu Z., Yang B., Gong W., Chen S., Wang S., Sun L., Song B., Xuan H. (2019). 3D printing of biomimetic vasculature for tissue regeneration. Mater. Horiz..

[12] Joshi S.C., Sheikh A.A. (2015). 3D printing in aerospace and its long-term sustainability. Virtual Phys. Prototyp..

[13] Maines E.M., Porwal M.K., Ellison C.J., Reineke T.M. (2021). Sustainable advances in SLA/DLP 3D printing materials and processes. Green Chem..

[14] Kantaros A., Diegel O., Piromalis D., Tsaramirsis G., Khadidos A.O., Khadidos A.O., Khan F.Q., Jan S. (2022). 3D printing: Making an innovative technology widely accessible through makerspaces and outsourced services. Mater. Today Proc..

[15] Sanchez-Rexach E., Johnston T.G., Jehanno C., Sardon H., Nelson A. (2020). Sustainable materials and chemical processes for additive manufacturing. Chem. Mater..

[16] Saadi M.A.S.R., Maguire A., Pottackal N.T., Thakur M.S.H., Ikram M.M., Hart A.J., Ajayan P.M., Rahman M.M. (2022). Direct ink writing: A 3D printing technology for diverse materials. Adv. Mater..

[17] Yadav D., Chhabra D., Garg R.K., Ahlawat A., Phogat A. (2020). Optimization of FDM 3D printing process parameters for multi-material using artificial neural network. Mater. Today Proc..

[18] Patel P., Dhal K., Gupta R., Tappa K., Rybicki F.J., Ravi P. (2023). Medical 3D printing using desktop inverted vat photopoly-merization: Background, clinical applications, and challenges. Bioengineering.

[19] Bagheri A., Jin J. (2019). Photopolymerization in 3D printing. ACS Appl. Polym. Mater.

[20] Hosseinabadi H.G., Nieto D., Yousefinejad A., Fattel H., Ionov L., Miri A.K. (2023). Ink material selection and optical design considerations in DLP 3D printing. Appl. Mater. Today.

[21] Sotov A., Kantyukov A., Popovich A., Sufiiarov V. (2021). LCD-SLA 3D printing of BaTiO_3_ piezoelectric ceramics. Ceram. Int..

[22] Wang Y., Li X., Chen Y., Zhang C. (2021). Strain rate dependent mechanical properties of 3D printed polymer materials using the DLP technique. Addit. Manuf..

[23] Colella R., Chietera F.P., Catarinucci L. (2021). Analysis of FDM and DLP 3D-printing technologies to prototype electromagnetic devices for RFID applications. Sensors.

[24] Saed A.B., Behravesh A.H., Hasannia S., Ardebili S.A.A., Akhoundi B., Pourghayoumi M. (2020). Functionalized poly l-lactic acid synthesis and optimization of process parameters for 3D printing of porous scaffolds via digital light processing (DLP) method. J. Manuf. Process..

[25] Peng B., Yang Y., Gu K., Amis E.J., Cavicchi K.A. (2019). Digital light processing 3D printing of triple shape memory polymer for sequential shape shifting. ACS Mater. Lett..

[26] Cometa S., Bonifacio M.A., Tranquillo E., Gloria A., Domingos M., De Giglio E. (2021). A 3D printed composite scaffold loaded with clodronate to regenerate osteoporotic bone: In vitro characterization. Polymers.

[27] Kreß S., Schaller-Ammann R., Feiel J., Priedl J., Kasper C., Egger D. (2020). 3D printing of cell culture devices: Assessment and prevention of the cytotoxicity of photopolymers for stereolithography. Materials.

[28] Li J.W., Lee J.C.M., Chuang K.C., Chiu C.W. (2023). Photocured, highly flexible, and stretchable 3D-printed graphene/polymer nanocomposites for electrocardiography and electromyography smart clothing. Prog. Org. Coat..

[29] Bagheri A., Bainbridge C.W.A., Engel K.E., Qiao G.G., Xu J., Boyer C., Jin J. (2020). Oxygen tolerant PET-RAFT facilitated 3D printing of polymeric materials under visible LEDs. ACS Appl. Polym. Mater.

[30] Browne M.P., Redondo E., Pumera M. (2020). 3D printing for electrochemical energy applications. Chem. Rev..

[31] Albar A., Chougan M., Al-Kheetan M.J., Swash M.R., Ghaffar S.H. (2020). Effective extrusion-based 3D printing system design for cementitious-based materials. Results Eng..

[32] Zhou Z., Qian C., Yuan W. (2021). Self-healing, anti-freezing, adhesive and remoldable hydrogel sensor with ion-liquid metal dual conductivity for biomimetic skin. Compos. Sci. Technol..

[33] Jia Z., Li Z., Ma S., Zhang W., Chen Y., Luo Y., Jia D., Zhong B., Razal J.M., Wang X. (2021). Constructing conductive titanium carbide nanosheet (MXene) network on polyurethane/polyacrylonitrile fibre framework for flexible strain sensor. J. Colloid Interface Sci..

[34] Chen D., Zhao X., Wei X., Zhang J., Wang D., Lu H., Jia P. (2020). Ultrastretchable, tough, antifreezing, and conductive cellulose hydrogel for wearable strain sensor. ACS Appl. Mater. Interfaces.

[35] Wang Z., Guan X., Huang H., Wang H., Lin W., Peng Z. (2019). Full 3D printing of stretchable piezoresistive sensor with hierarchical porosity and multimodulus architecture. Adv. Funct. Mater..

[36] Lin C.L., Li J.W., Chen Y.F., Chen J.X., Cheng C.C., Chiu C.W. (2022). Graphene nanoplatelet/multiwalled carbon nanotube/polypyrrole hybrid fillers in polyurethane nanohybrids with 3D conductive networks for EMI shielding. ACS Omega.

[37] Men Q., Wang S., Yan Z., Zhao B., Guan L., Chen G., Guo X., Zhang R., Che R. (2022). Iron-encapsulated CNTs on carbon fiber with high-performance EMI shielding and electrocatalytic activity. Adv. Compos. Hybrid Mater..

[38] Jia Z., Zhang X., Gu Z., Wu G. (2023). MOF-derived Ni-Co bimetal/porous carbon composites as electromagnetic wave absorber. Adv. Compos. Hybrid Mater..

[39] Ai L., Li S., Cao H., Zhu Y. (2023). Conductive properties of polyester/spandex fabrics using liquid carbon black and disperse black dye. ACS Omega.

[40] Xu Q., Wu Z., Zhao W., He M., Guo N., Weng L., Lin Z., Abou Taleb M.F., Ibrahim M.M., Singh M.V. (2023). Strategies in the preparation of conductive polyvinyl alcohol hydrogels for applications in flexible strain sensors, flexible supercapacitors, and triboelectric nanogenerator sensors: An overview. Adv. Compos. Hybrid Mater..

[41] Dong H., Sun J., Liu X., Jiang X., Lu S. (2022). Highly sensitive and stretchable MXene/CNTs/TPU composite strain sensor with bilayer conductive structure for human motion detection. ACS Appl. Mater. Interfaces.

[42] Tas M.O., Baker M.A., Masteghin M.G., Bentz J., Boxshall K., Stolojan V. (2019). Highly stretchable, directionally oriented carbon nanotube/PDMS conductive films with enhanced sensitivity as wearable strain sensors. ACS Appl. Mater. Interfaces.

[43] Anjos E.G.R., Brazil T.R., Melo Morgado G.F., Antonelli E., Rezende M.C., Pessan L.A., Moreira F.K.V., Marini J., Passador F.R. (2023). Renewable PLA/PHBV blend-based graphene nanoplatelets and carbon nanotube hybrid nanocomposites for electromagnetic and electric-related applications. ACS Appl. Electron. Mater..

[44] Cai Y., Yu H., Chen C., Feng Y., Qin M., Feng W. (2022). Improved thermal conductivities of vertically aligned carbon nanotube arrays using three-dimensional carbon nanotube networks. Carbon.

[45] Wang J., Zhang X., Liu Y., Xu C., Zhang H., Wu D., Tan T., Qin X., Sun J., Zhang L. (2021). Preparation of flexible and elastic thermal conductive nanocomposites via ultrasonic-assisted forced infiltration. Compos. Sci. Technol..

[46] Mora A., Verma P., Kumar S. (2020). Electrical conductivity of CNT/polymer composites: 3D printing, measurements and modeling. Compos. B Eng..

[47] Chiu C.W., Lin J.J. (2012). Self-assembly behavior of polymer-assisted clays. Prog. Polym. Sci..

[48] (2014). Standard Test Method for Tensile Properties of Plastics.

