# Supplementary material for: Photocurable Carbon Nanotube/Polymer Nanocomposite for the 3D Printing of Flexible Capacitive Pressure Sensors
Source: Polymers (Basel). 2023 Dec 14;15(24):4706. doi: 10.3390/polym15244706 (PMC10747156; doi:10.3390/polym15244706)
Supplement: Supplementary file 1 [file polymers-15-04706-s001.zip › polymers-2757400-supplementary.pdf]

polymers

MDPI

Article

Photocurable Carbon Nanotube/Polymer Nanocomposite for the 3D Printing of Flexible Capacitive Pressure Sensors

Jia-Wun Li [1,†], Ho-Fu Chen [1,†], Peng-Han Huang [1,†], Chung-Feng Jeffrey Kuo [1], Chih-Chia Cheng [2] and Chih-Wei Chiu [1,*]

[1] Department of Materials Science and Engineering, National Taiwan University of Science and Technology, Taipei 10607, Taiwan
[2] Graduate Institute of Applied Science and Technology, National Taiwan University of Science and Technology, Taipei 10607, Taiwan
* Correspondence: cwchiu@mail.ntust.edu.tw; Tel.: +886-2-2737-6521
† These authors contributed equally to this work.

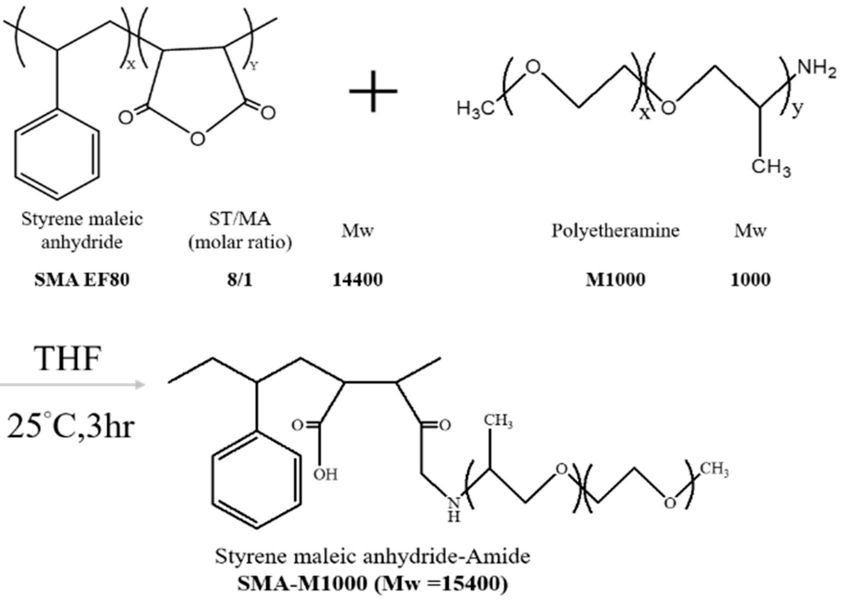

Figure S1. Synthesis of SMA-M1000 polymeric dispersant.

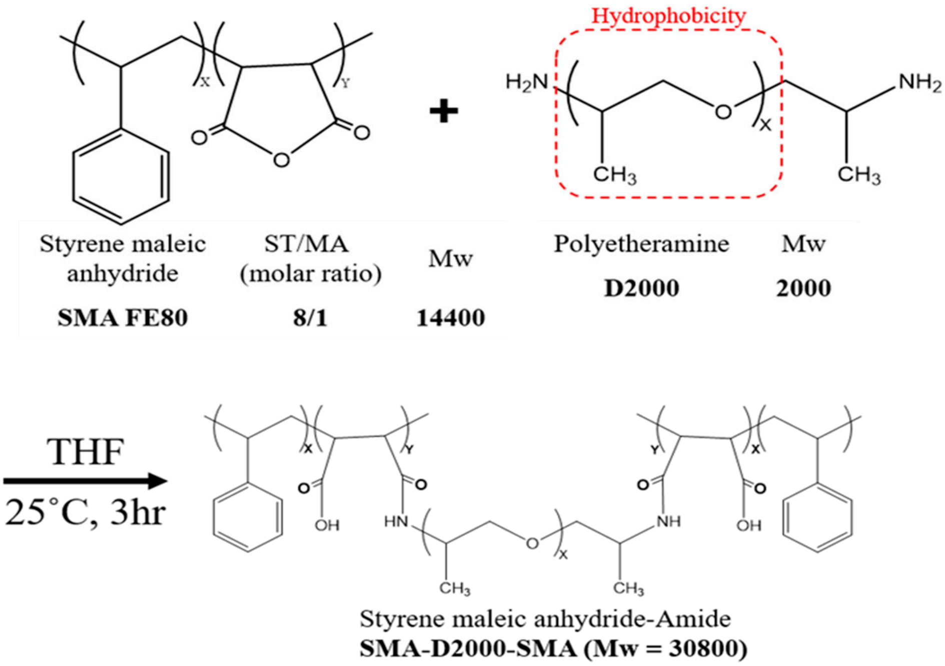

Figure S2. Synthesis of SMA-D2000 polymeric dispersant.

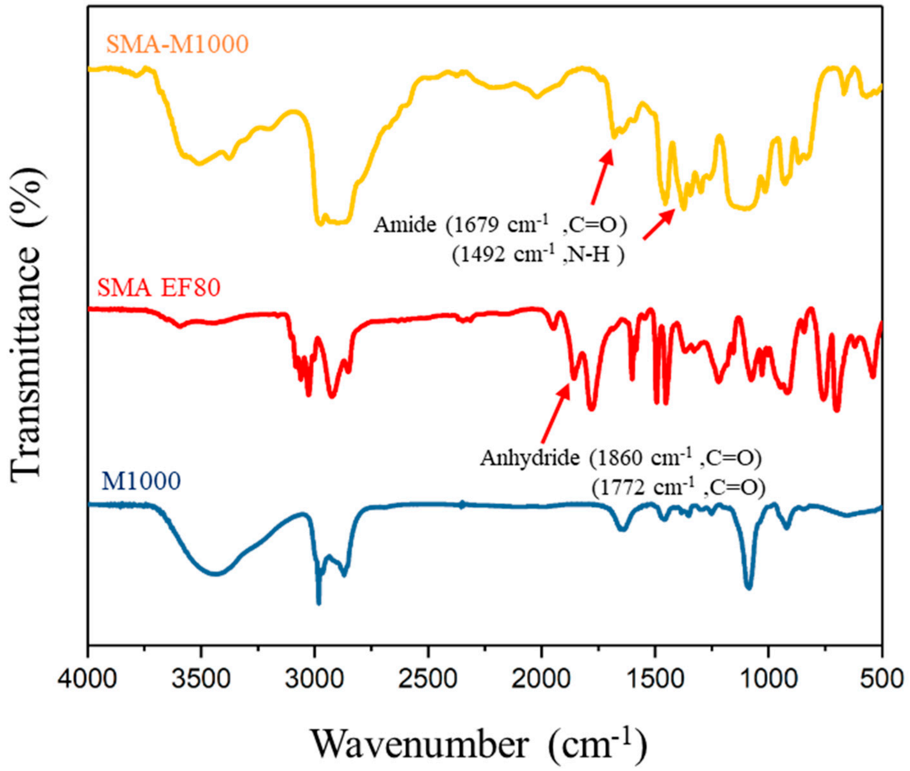

Figure S3. FT-IR analysis of M1000, SMA EF80, and SMA-M1000.

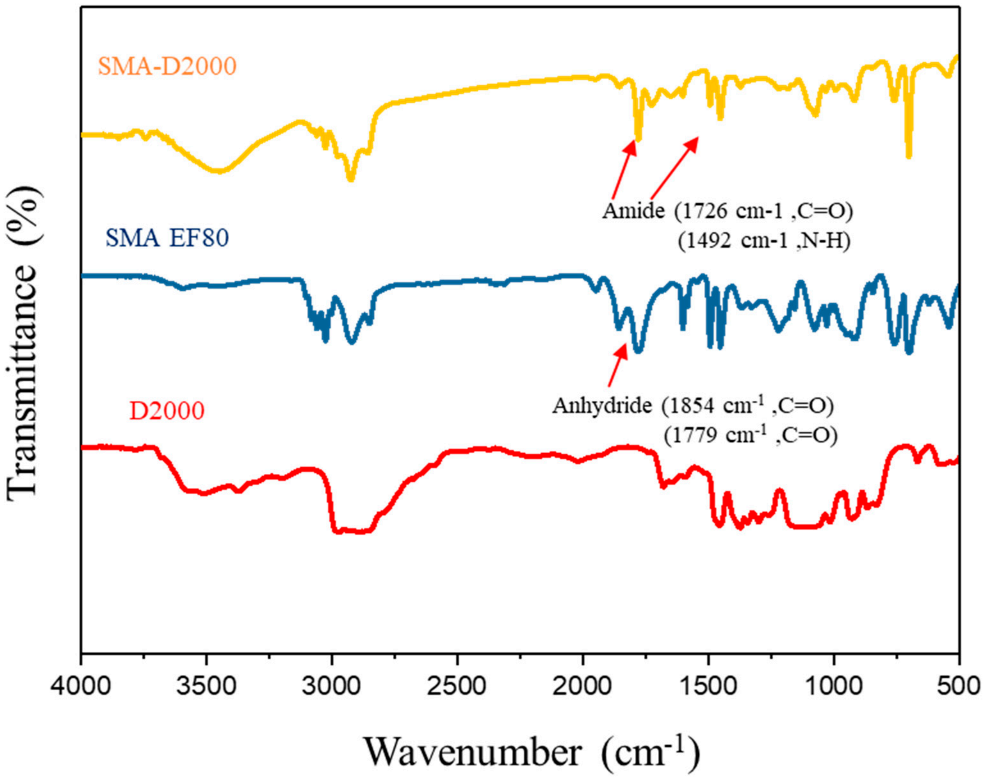

Figure S4. FT-IR spectra of D2000, SMA EF80, and SMA-D2000.

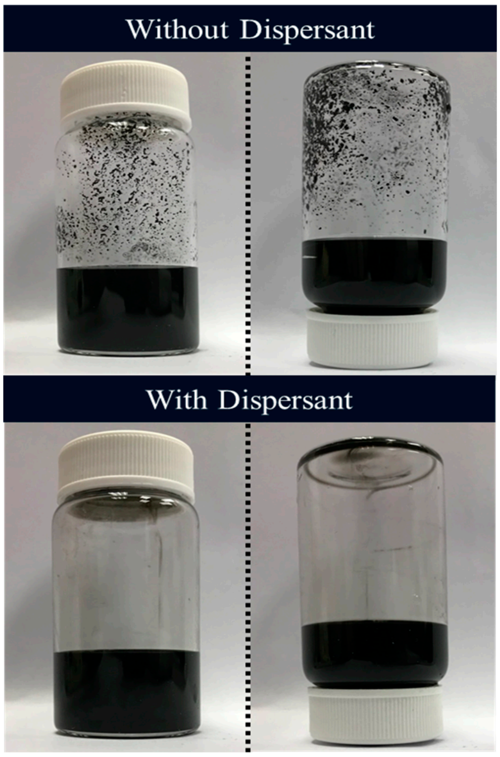

Figure S5. Photographs of CNT dispersion before and after adding polymeric dispersants.

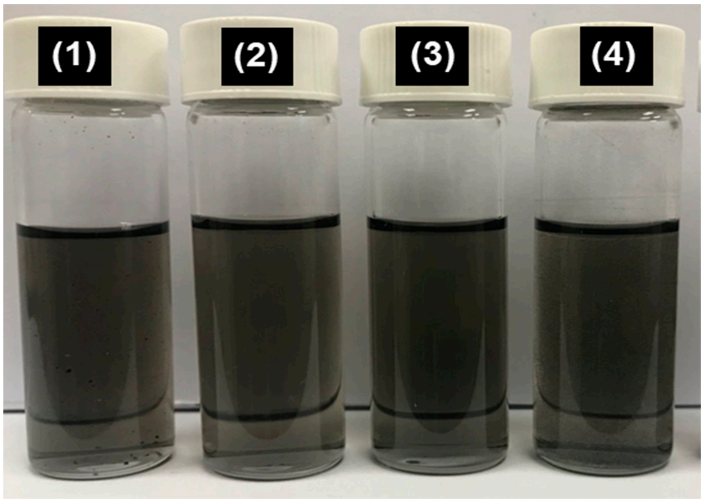

Figure S6. Photographs of CNT/polymer dispersions immediately after preparation: (1) without dispersant, (2) EF80, (3) M-1000, and (4) SMA-M1000.

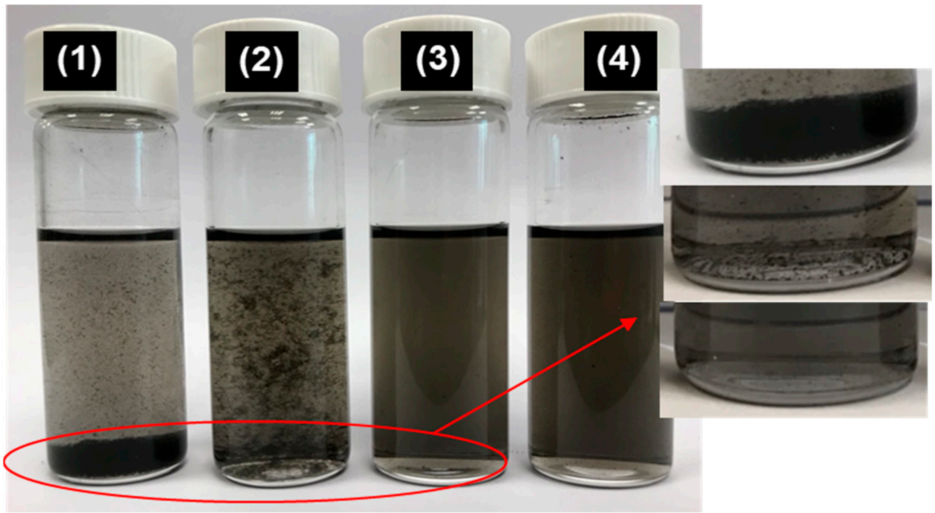

Figure S7. Photographs of CNTs/polymer dispersions five days after preparation: (1) without dispersant, (2) EF80, (3) M-1000, and (4) SMA-M1000.

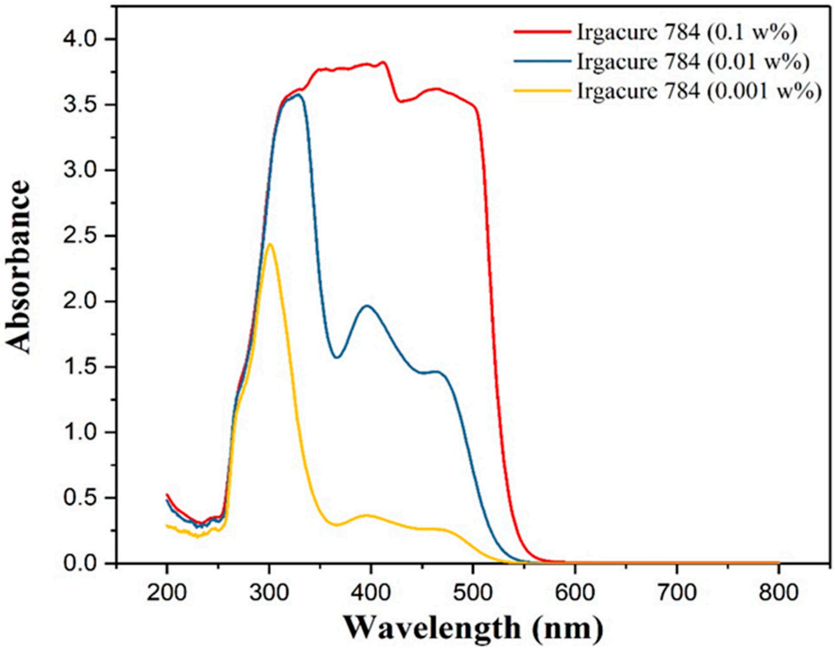

Figure S8. UV-vis spectrum of the photoinitiator.

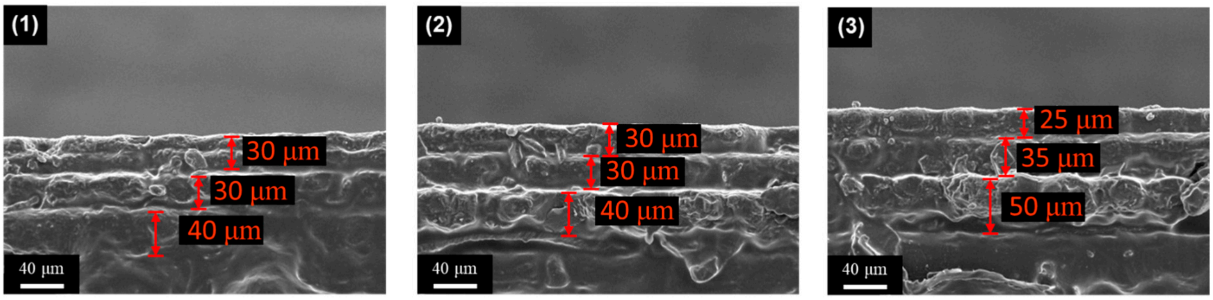

Figure S9. FE-SEM observation of the cross-section of 3D-printed objects with different layer heights: (1) 30 µm, (2) 40 µm, and (3) 50 µm.

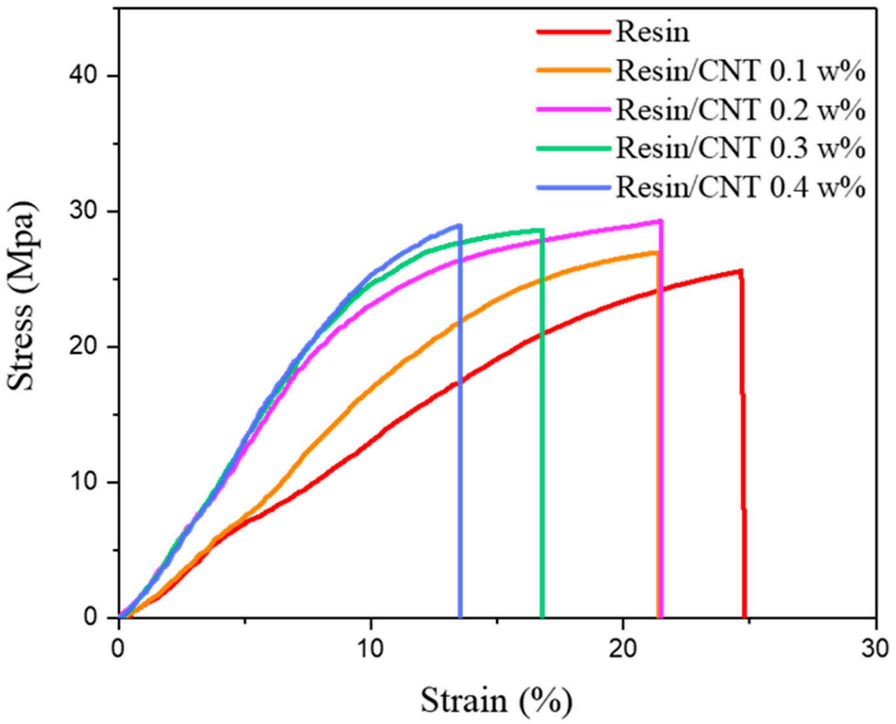

Figure S10. Stress–strain curves of CNT/resin nanocomposites without dispersant.

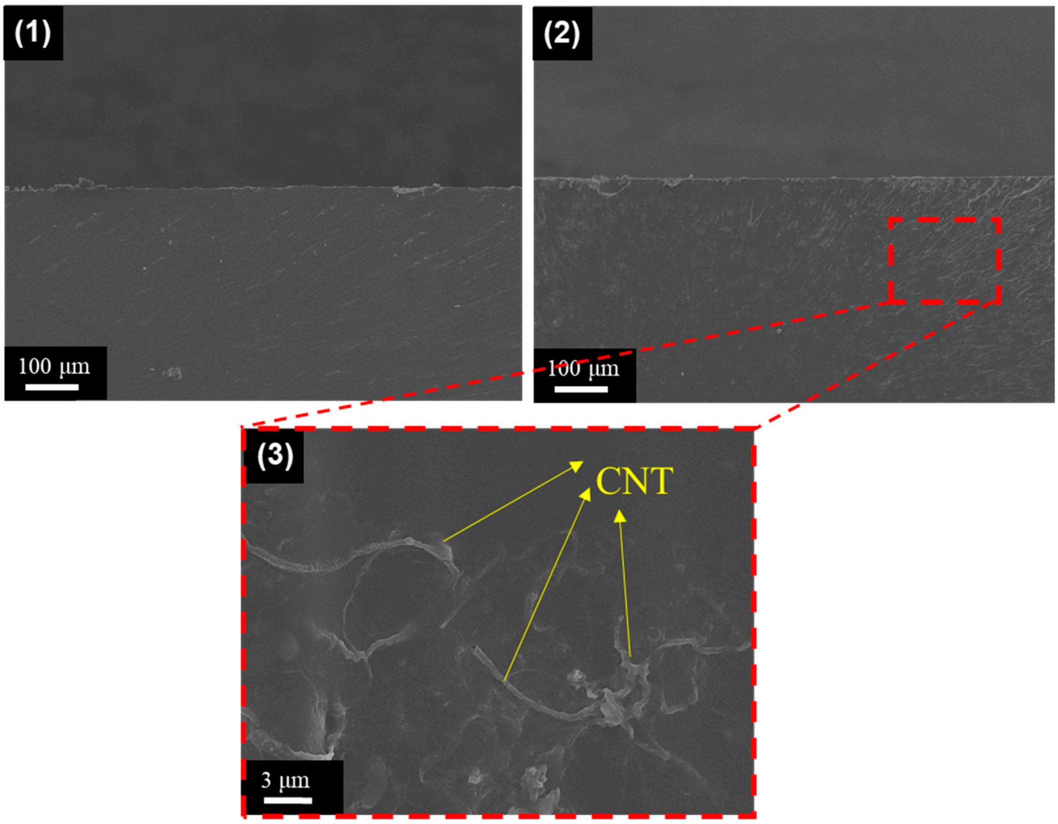

Figure S11. FE-SEM micrographs of the fracture surface of CNT/resin nanocomposites after tensile failure. (1) Pure resin (without CNT); (2) nanocomposite with 0.3 wt% CNT; (3) magnified view of an area in (2).

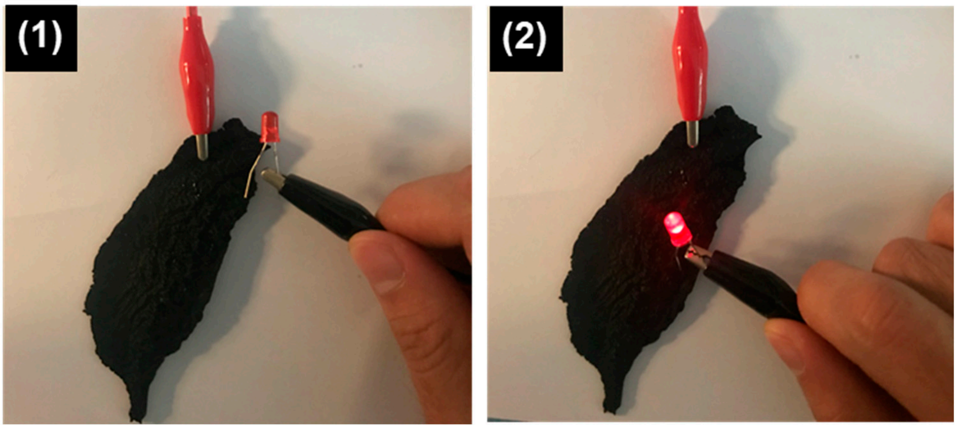

Figure S12. Demonstration of the conductivity of an object fabricated via DLP 3D-printing with the developed photocurable resin/CNT nanocomposite.

```
#include <CapacitiveSensor.h>

CapacitiveSensor cs1 = CapacitiveSensor(6,7);
CapacitiveSensor cs2 = CapacitiveSensor(6,5);
int ledPin = 2;
int ledPin2 = 3;
void setup() {
Serial.begin(9600); // put your setup code here, to run once:
pinMode(ledPin, OUTPUT);
pinMode(ledPin2, OUTPUT);
}

void loop() {
  // put your main code here, to run repeatedly:
long cs1v = cs1.capacitiveSensor(80);
long cs2v = cs2.capacitiveSensor(80);

if(cs1v>300)
  digitalWrite(ledPin, HIGH);
else
  digitalWrite(ledPin, LOW);

if(cs2v>300)
  digitalWrite(ledPin2, HIGH);
else
  digitalWrite(ledPin2, LOW);

Serial.print(cs1v);
Serial.print(" ");
Serial.println(cs2v);
delay(100);
}
```

Figure S13. Arduino code for the capacitive pressure sensor.

Table S1. Solubility of the polymeric dispersants in different solvents.

	**Polar Solvents** →						**Non-Polar Solvents**
Solvent	H_2O	DMF	NMP	EtOH	MEK	THF	Toluene
SMA EF80	-	+	+	-	+	+	-
M000	+	+	+	+	+	+	+
D2000	-	+	+	+	+	+	+
SMA-M1000	+/-	+	+	+	+	+	+
SMA-D2000	-	+	+	+	+	+	+

+: soluble; +/-: soluble but forming sediments after 2 h settlement; -: insoluble.
